# Mixotrophic Cultivation of *Limnospira* (*Spirulina*) *platensis* Using Early-Stage Fig Processing Wastewater: Effects on Biomass Composition, Antioxidants and Phycocyanin

**DOI:** 10.3390/md24050163

**Published:** 2026-05-05

**Authors:** Luca Franzoso, Luca Usai, Riccardo Allodi, Giacomo Fais, Deborah Dessì, Robinson Soto-Ramirez, Bartolomeo Cosenza, Abderrahim Damergi, Giovanni Antonio Lutzu, Alessandro Concas

**Affiliations:** 1 Department of Life Sciences, University of Modena and Reggio Emilia, via Giuseppe Campi 287, 41123 Modena, MO, Italy; 2 Teregroup Srl, via David Livingstone 37, 41123 Modena, MO, Italygianni.lutzu@teregroup.net (G.A.L.); 3 Department of Mechanical, Chemical and Materials Engineering, University of Cagliari, Piazza d’Armi, 09123 Cagliari, CA, Italy; 4 Interdepartmental Center of Environmental Science and Engineering (CINSA), University of Cagliari, via San Giorgio 12, 09124 Cagliari, CA, Italy; 5 Department of Life and Environmental Sciences, University of Cagliari, Cittadella Universitaria, Blocco A, SP8 Km 0.700, 09042 Monserrato, CA, Italy; 6 Departamento de Ingeniería de Procesos y Bioproductos, Facultad de Ingeniería, Universidad del Bio-Bio, Avda. Collao 1202, Concepción 4051381, Region del Bio-Bio, Chile; 7 Department of Civil and Industrial Engineering, University of Pisa, Largo Lucio Lazzarino, 56122 Pisa, PI, Italy; 8 AlgaStream LLC R&D, C1 Imm Haouel, Avenue de la République, Hammamet 8050, Tunisia; rahim.damergi@alga-stream.com

**Keywords:** *Limnospira platensis*, circular bioeconomy, cyanobacterial biotechnology, agro-industrial residue valorization, phycobiliprotein production, antioxidant metabolites

## Abstract

The valorization of agro-industrial waste streams represents a promising strategy for reducing production costs in microalgae biotechnology while promoting circular economy approaches. In this study, wastewater derived from fig jam processing was evaluated as an organic carbon source for mixotrophic cultivation of *Limnospira* (*Spirulina*) *platensis*. Cultures were grown under four conditions: a control medium and three concentrations of fig wastewater (FW) at 0.75%, 1.5%, and 3% (*v v*^−1^). The wastewater used in this study originates specifically from the washing and cleaning stages of dried fig processing, representing an early processing stream characterized by relatively high soluble sugar content and low thermal or chemical alteration. Biomass biochemical composition and bioactive compound production were investigated, including carbohydrates, proteins, lipids, photosynthetic pigments, polyphenols, antioxidant activity, and phycocyanin extraction yield and purity. The results showed that fig wastewater supplementation significantly influenced the metabolic profile of *L. platensis*. The highest protein content was obtained at 0.75% FW (44.90 ± 1.93 g 100 g^−1^ DW), whereas lipid accumulation increased with FW concentration, reaching 9.45 ± 2.30 g 100 g^−1^ DW at 3% FW. Antioxidant activity peaked at 1.5% FW (4.33 ± 0.43 μmol Trolox mg^−1^ DW), suggesting stimulation of oxidative stress response pathways under moderate organic supplementation. Pigment production showed different responses, with relatively stable chlorophyll and carotenoid contents but decreasing phycocyanin levels at higher FW concentrations. Phycocyanin yield decreased from 9.82 ± 1.00 g 100 g^−1^ DW in the control to 5.80 ± 0.22 g 100 g^−1^ DW at 3% FW, while purity values were highest at the highest FW concentration. These findings demonstrate that fig processing wastewater can be effectively used as an alternative organic substrate for mixotrophic *Spirulina* cultivation, enabling simultaneous wastewater valorization and production of biomass rich in proteins and bioactive compounds.

## 1. Introduction

Microalgae and cyanobacteria have attracted increasing attention as sustainable biological platforms for the production of high-value biomolecules, including proteins, pigments, antioxidants, and polyunsaturated lipids [1]. Among them, the filamentous cyanobacterium *Limnospira* (*Spirulina*) *platensis* is one of the most extensively cultivated microorganisms worldwide due to its remarkable nutritional profile and its capacity to produce bioactive compounds with applications in food, pharmaceutical, and cosmetic industries [2,3]. In particular, *L. platensis* biomass is characterized by a high protein content, often exceeding 50–60% of dry weight, as well as by the presence of valuable pigments such as phycocyanin (PC), chlorophylls, and carotenoids, which exhibit antioxidant, anti-inflammatory, and nutraceutical properties [4,5].

Despite its commercial relevance, large-scale cultivation of *L. platensis* remains constrained by the high costs associated with mineral culture media and operational requirements. In this context, alternative cultivation strategies such as the use of seawater and mixotrophic systems that combine photosynthetic metabolism with the assimilation of organic carbon sources, have been widely explored to improve biomass productivity while reducing production costs [6,7,8].

Microalgal growth can occur under different trophic modes, including autotrophic, heterotrophic, and mixotrophic conditions, each characterized by distinct carbon and energy acquisition mechanisms. Under autotrophic conditions, microalgae rely exclusively on inorganic carbon (CO_2_ or HCO_3_^−^) and light energy, converting them into biomass through photosynthesis. In contrast, heterotrophic growth occurs in the absence of light, where organic carbon sources are metabolized to provide both energy and carbon skeletons, although this mode is limited in many cyanobacteria, including *L. platensis*, due to their intrinsic dependence on photosynthetic metabolism. Mixotrophic cultivation combines these two modes, allowing simultaneous utilization of inorganic carbon through photosynthesis and organic substrates through heterotrophic pathways. This dual metabolism can enhance metabolic flexibility by increasing the availability of reducing equivalents and ATP, thereby supporting higher biosynthetic activity and, in some cases, improved production of bioactive compounds [9,10].

The assimilation of organic carbon in mixotrophic systems depends on the ability of microalgae to uptake and metabolize soluble organic compounds, particularly simple sugars such as glucose and fructose, which are commonly found in agro-industrial effluents. These sugars are transported into the cell via specific membrane transport systems and are subsequently metabolized through central carbon pathways, including glycolysis and the oxidative pentose phosphate pathway. These metabolic routes generate intermediates for biomass synthesis as well as reducing power (e.g., NADPH), which plays a key role in anabolic processes and cellular redox balance [9,10].

In the case of complex or macromolecular carbohydrates, extracellular or periplasmic hydrolysis may be required to convert them into assimilable monomers prior to uptake. The efficiency of these processes depends on both the composition of the substrate and the metabolic capabilities of the microorganism. Therefore, the presence of readily assimilable sugars in agro-industrial wastewaters represents a critical factor influencing the performance of mixotrophic cultivation systems [11].

The use of agro-industrial residues as substrates for microalgal cultivation represents a promising strategy within the framework of circular bioeconomy [12,13]. Many agro-food industries generate waste streams rich in organic carbon, sugars, and micronutrients that could be effectively exploited as growth media for microalgae [5,7,14]. The reuse of such residues not only reduces the environmental burden associated with wastewater disposal but also enables the recovery of valuable resources through biological processes [15].

Among fruit-processing sectors, the dried fig (*Ficus carica* L.) industry represents an important agro-industrial activity, particularly in Mediterranean countries such as Turkey, Egypt, Morocco, Greece, Italy, and Spain, which together account for the majority of global production [16,17]. According to recent agricultural statistics, global fig production exceeds one million tons per year, with a substantial fraction being processed and commercialized as dried fruits or transformed into value-added products such as jams, syrups, bakery ingredients, and confectionery preparations [18]. The processing of dried figs therefore generates several types of organic residues and process waters that require appropriate management.

In industrial processing lines, dried figs typically undergo preliminary treatments prior to further transformation. These steps commonly include rehydration, washing, and cleaning of the dried fruits in order to remove dust, impurities, and surface contaminants accumulated during drying, handling, and storage [19]. These operations generate considerable volumes of process water enriched with soluble organic compounds released from the fruit matrix, including simple sugars, organic acids, phenolic compounds, and micronutrients [20].

Dried figs are naturally rich in carbohydrates, with total sugar contents typically exceeding 50–60% of dry weight [17]. The predominant sugars are glucose and fructose, which together account for the majority of soluble carbohydrates in the fruit [21]. During hydration and washing steps, part of these sugars can leach into the process water, leading to the formation of sugar-rich effluents characterized by elevated biochemical oxygen demand (BOD) and chemical oxygen demand (COD) [22]. Although these waters are generally considered waste streams requiring treatment prior to disposal, their composition suggests that they may represent a valuable resource for microbial or microalgal cultivation [23].

In recent years, increasing attention has been directed toward the integration of agro-industrial residues into microalgal biorefinery systems, where waste streams are used as nutrient sources for microalgal growth while simultaneously generating valuable biomass [24]. Within this concept, microalgae can convert organic-rich effluents into a range of bioactive compounds, including pigments, antioxidants, proteins, and lipids [25]. Such integrated approaches allow the coupling of wastewater remediation with the production of high-value biomolecules, thereby improving process sustainability and economic feasibility.

Fruit-derived residues have already been explored as substrates for microalgal cultivation due to their high content of readily assimilable sugars and organic nutrients. However, the potential of process waters generated during the washing and cleaning of dried figs has received limited attention in the context of microalgal biotechnology.

Because agro-industrial process waters may simultaneously provide readily assimilable nutrients and impose compositional stress, the response of *L. platensis* to fig wastewater (FW) supplementation was interpreted not only in terms of growth promotion, but also in relation to potential stress-mediated effects on pigment synthesis and antioxidant metabolism.

Therefore, the aim of this study was to evaluate the feasibility of using process water generated during the washing and cleaning of dried figs as an organic supplement for the mixotrophic cultivation of *L. platensis*. The effects of increasing concentrations of this wastewater on biomass biochemical composition, pigment synthesis, antioxidant activity, and PC extraction were investigated in order to assess the potential of this agro-industrial effluent as a sustainable substrate for microalgal cultivation and the production of bioactive compounds. In addition to mixotrophic cultivation, heterotrophic conditions were included as a control to decouple the effects of organic carbon supplementation from light-driven photosynthesis, allowing a clearer assessment of the metabolic contribution of FW.

## 2. Results and Discussion

### 2.1. Growth Profile of Limnospira platensis Under FW Supplementation

**Figure 1:**
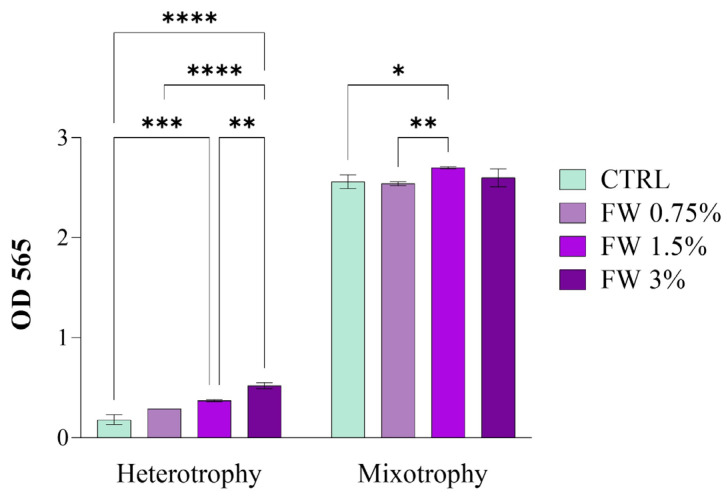
Optical density (OD_565_) of *Limnospira platensis* cultures after 8 days of batch cultivation under different trophic conditions: CTRL = control Jourdan medium, FW 0.75% = CTRL + 0.75% FW, FW 1.5% = CTRL + 1.5% FW, FW3 = CTRL + 3% FW. Values represent mean ± standard deviation (*n* = 3 biological replicates). Statistical differences were assessed by two-way ANOVA with Tukey’s post hoc test; different asterisks above bars indicate significant differences (* *p* < 0.05, ** *p* < 0.01, *** *p* < 0.001, **** *p* < 0.0001). OD measurements provide a comparative indicator of growth but do not capture lag phase duration or detailed growth kinetics.

The growth of *Limnospira platensis* was evaluated in batch cultures for eight days using FW as an organic carbon source under both mixotrophic and heterotrophic conditions. FW was tested at 0.75%, 1.5%, and 3% (*v v*^−1^) in the modified JM. The objective was to assess how increasing FW concentrations affect key kinetic parameters, including final biomass concentration (X_f_), biomass increment (ΔX), volumetric productivity (Q_x_), average specific growth rate (μ), and doubling time (t_d_) (Table 1). Growth dynamics were monitored through optical density measurements (OD_565_), which provide a comparative indicator of biomass accumulation (Figure 1).

Under mixotrophic conditions (where both light and organic carbon are available), FW supplementation did not drastically alter the overall growth pattern compared to the control culture. As shown in Table 1, the final biomass concentration ranged between 1.97 and 2.10 g L^−1^, indicating that the addition of FW within the tested range did not significantly impair culture performance. The highest biomass concentration (2.10 ± 0.01 g L^−1^) was recorded at 1.5% FW, suggesting that this concentration may provide a favorable balance between inorganic and organic carbon assimilation. A similar trend was observed for volumetric productivity, which increased slightly from 0.29 g L^−1^ day^−1^ in the control to 0.31 g L^−1^ day^−1^ in FW-supplemented cultures.

The average specific growth rate (μ) remained relatively stable across treatments, varying between 0.19 and 0.21 day^−1^, indicating that FW supplementation did not significantly alter the intrinsic growth capacity of the culture under illuminated conditions. However, a slight improvement in culture kinetics was observed at higher FW concentrations, as reflected by the shorter doubling time, which decreased from 3.68 days in the control to 3.33 days at 3% FW. These results suggest that moderate FW supplementation can modestly enhance biomass turnover without substantially affecting metabolic stability.

The response observed under heterotrophic conditions differed markedly from that observed in mixotrophic cultures. In the absence of light, FW acted as the sole carbon and energy source, resulting in a clear dose-dependent increase in biomass accumulation (Table 1).

Final biomass concentration increased progressively from 0.14 g L^−1^ in the control to 0.40 g L^−1^ at 3% FW, representing nearly a threefold increase. A similar trend was observed for volumetric productivity, which rose from 0.07 g L^−1^ day^−1^ in the control to 0.28 g L^−1^ day^−1^ at 3% FW. The kinetic parameters further support this trend. The specific growth rate increased steadily from 0.62 day^−1^ in the control to 1.25 day^−1^ at 3% FW, while the doubling time decreased from 1.14 to 0.55 days, indicating a substantial improvement in growth kinetics as FW concentration increased.

This behavior highlights the ability of *L. platensis* to utilize organic substrates present in FW under dark conditions, although biomass yields remained significantly lower than under mixotrophy due to the absence of photosynthetic carbon fixation.

Broadly, under dark conditions, cultures supplemented with 0.75–3% FW exhibited slower growth compared to the mixotrophic setups, which aligns with previous studies where organic carbon supplementation under heterotrophy often leads to lower growth rates compared to mixotrophic conditions [26]. In the control heterotrophic culture, where no FW was added, growth was minimal, demonstrating the limited capacity for pure heterotrophic growth in *L. platensis*.

The inclusion of heterotrophic conditions in this study provides important insight into the role of organic carbon in supporting *L. platensis* metabolism. The markedly lower biomass concentrations observed under dark conditions, despite increasing FW supplementation, indicate that this cyanobacterium has a limited capacity to sustain growth solely on external organic carbon sources. This behavior is consistent with the known physiology of *L. platensis*, which is primarily a photoautotrophic organism and exhibits restricted heterotrophic growth unless specific adaptation strategies are applied [27].

Accordingly, the reduced productivity observed under heterotrophic conditions was expected and should not be interpreted as a limitation of FW itself. Rather, these results demonstrate that FW alone is insufficient to sustain efficient growth and confirm that the enhanced performance observed under mixotrophic conditions arises from the synergistic interaction between photosynthesis and organic carbon assimilation. In this context, heterotrophic cultivation should be regarded as a reference condition used to decouple the contribution of organic carbon from light-driven metabolism, rather than as a viable alternative cultivation strategy.

The pH dynamics observed during cultivation also reflected the metabolic activity of the cultures. Under mixotrophic conditions, pH values stabilized around 10.6–10.8, which is consistent with the typical alkalinity of *L. platensis* cultures and reflects CO_2_ consumption during photosynthesis. Conversely, heterotrophic cultures maintained a slightly lower pH (9.64–9.71), likely due to the absence of photosynthetic CO_2_ uptake and the accumulation of metabolic by-products. Such pH behavior is commonly observed in cyanobacterial cultures and can influence nutrient availability and metabolic activity [28,29].

Overall, these results indicate that FW supplementation within the range of 0.75–3% does not negatively affect the growth of *L. platensis* under mixotrophic conditions, while under heterotrophy it significantly enhances biomass production and growth kinetics. Among the tested conditions, 1.5% FW provided the best compromise between biomass accumulation and kinetic performance under mixotrophy, whereas 3% FW yielded the highest biomass under heterotrophic conditions. These findings support the potential use of FW as a sustainable organic substrate for microalgal cultivation, contributing to the valorization of agro-industrial residues within circular bioeconomy strategies.

Despite enhanced biomass accumulation under heterotrophy, the rationale for selecting mixotrophic cultivation for subsequent biochemical analyses lies in the substantially higher biomass productivity obtained under illuminated conditions. In the present study, *L. platensis* cultures grown under mixotrophy exhibited a 5–15-fold increase in biomass accumulation compared to heterotrophy (Table 1), clearly indicating that photosynthetic metabolism remains the dominant pathway sustaining cellular growth in this cyanobacterium. Although *L. platensis* can assimilate organic carbon sources under dark conditions, previous studies have demonstrated that its heterotrophic growth capacity is intrinsically limited, primarily due to its metabolic dependence on photosynthetic carbon fixation and its relatively inefficient uptake and assimilation of exogenous organic substrates [30,31]. Consequently, heterotrophic cultures typically produce lower biomass yields and reduced pigment accumulation compared to mixotrophic or photoautotrophic conditions.

Because most biochemical analyses, such as pigment quantification, antioxidant activity, and proximate composition, require sufficient biomass availability to ensure analytical robustness and reproducibility, mixotrophic cultivation provided a more suitable experimental platform. The significantly higher biomass yield obtained under these conditions ensured that the biochemical characterization of the cultures could be conducted reliably while also reflecting physiologically relevant metabolic states.

### 2.2. Pigments and Antioxidants Content of Limnospira platensis Under FW Cultivation

**Figure 2:**
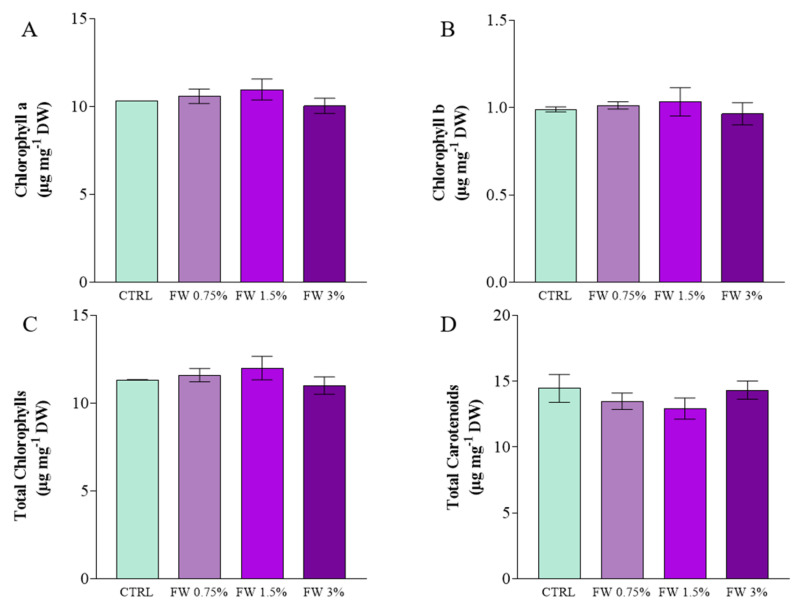
Photosynthetic pigment content in *L. platensis* cultivated under mixotrophic conditions with different concentrations of fig wastewater (FW: 0.75%, 1.5%, and 3% *v v*^−1^). (**A**) Chlorophyll a; (**B**) Chlorophyll b; (**C**) Total Chlorophylls, (**D**) Total Carotenoids. Data represent mean ± standard deviation of triplicate experiments. Statistical analysis was performed using one-way ANOVA followed by Tukey’s post hoc test; no statistically significant differences were observed among treatments (*p* > 0.05).

**Figure 3:**
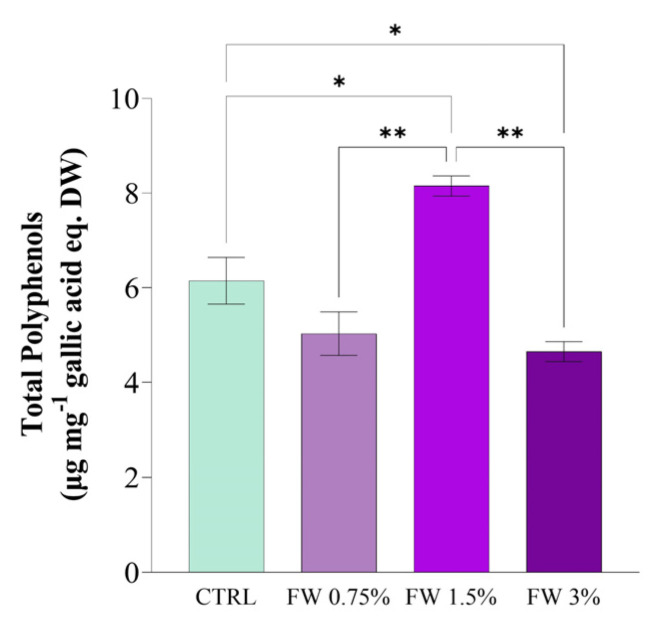
Total polyphenol content in *L. platensis* biomass cultivated under mixotrophic conditions with different concentrations of fig wastewater (FW: 0.75%, 1.5%, and 3% *v v*^−1^). Results are expressed as μg gallic acid equivalents (GAE) mg^−1^ dry weight. Values represent mean ± SD (*n* = 3). Mean differences were compared using Tukey’s test (*n* = 3, * *p* < 0.05, ** *p* < 0.01).

**Figure 4:**
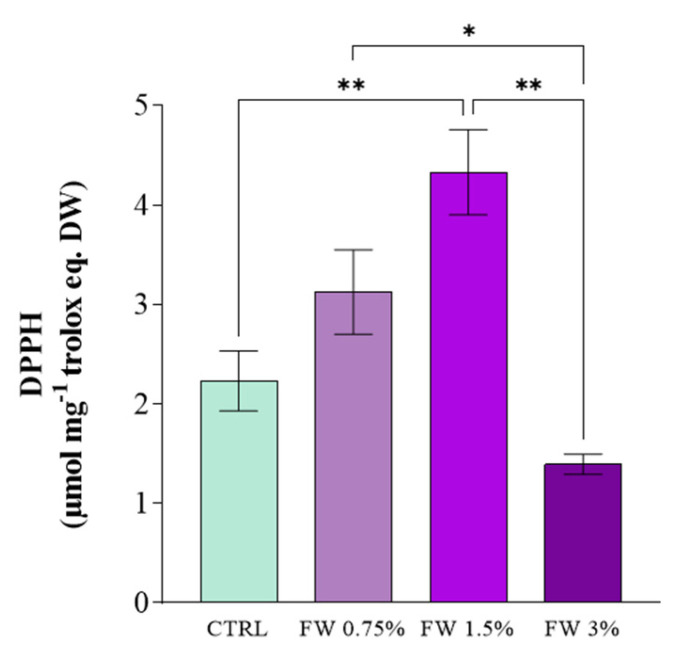
Antioxidant activity of *L. platensis* extracts determined by DPPH radical scavenging assay obtained under mixotrophic conditions with different concentrations of fig wastewater (FW: 0.75%, 1.5%, and 3% *v v*^−1^). Results are expressed as μmol Trolox equivalents mg^−1^ dry weight. Data represent mean ± SD (*n* = 3). Mean differences were compared using Tukey’s test (*n* = 3, * *p* < 0.05, ** *p* < 0.01).

The supplementation of the culture medium with FW significantly influenced the metabolic profile of *L. platensis*, particularly affecting pigment composition and antioxidant-related metabolites (Figure 2, Figure 3 and Figure 4). FW generated during the washing and processing of dried figs contains soluble organic compounds originating from the fruit matrix, including simple sugars (Table 2), organic acids, and phenolic compounds. These substrates can act as additional carbon and energy sources for cyanobacterial metabolism, thereby influencing cellular physiology and the synthesis of bioactive metabolites [9].

As shown in Figure 2, moderate FW supplementation slightly increased the concentration of photosynthetic pigments. Chlorophyll *a* content increased from the control condition (~10.3 µg mg^−1^ DW) to a maximum value at 1.5% FW (~11 µg mg^−1^ DW) (Figure 2A). A comparable trend was observed for total chlorophylls (Figure 2C), suggesting that moderate FW supplementation supports photosynthetic metabolism without inducing physiological stress. The presence of organic carbon sources may improve the cellular energy balance by providing additional reducing equivalents and ATP, thereby promoting pigment biosynthesis and cellular growth under mixotrophic conditions. Similar enhancements in chlorophyll production under organic carbon supplementation have been reported in cyanobacteria and microalgae cultivated under mixotrophic regimes [10,26].

Chlorophyll *b* values were also reported (Figure 2B) although *L. platensis*, as a cyanobacterium, does not naturally synthesize this pigment. The absorbance signal detected at 649 nm is therefore likely attributable to spectral interference from accessory pigments or degradation products. Similar methodological considerations have been reported in previous spectrophotometric studies where Arnon’s equations were applied for comparative purposes [32].

Interestingly, carotenoid levels showed an inverse trend relative to chlorophylls (Figure 2D). While the control culture exhibited the highest carotenoid concentration (~14.5 µg mg^−1^ DW), the addition of FW led to a slight reduction at intermediate concentrations, followed by a recovery at 3% FW. Carotenoids play a crucial photoprotective role in cyanobacteria by dissipating excess energy and mitigating oxidative stress generated during photosynthesis. Variations in carotenoid content may therefore reflect physiological adjustments to changes in carbon availability and metabolic fluxes induced by organic supplementation. Similar pigment modulation has been observed in microalgae cultivated under mixotrophic conditions where organic carbon reduces the reliance on photosynthetic energy metabolism [33]. In the present study, the modest decrease observed at intermediate FW concentrations may indicate a reduction in photooxidative stress due to the additional metabolic flexibility provided by organic carbon.

It should be noted that the addition of FW may have slightly altered the optical properties of the culture medium, particularly at higher concentrations. However, the wastewater used in this study was filtered and derived from early processing stages, resulting in relatively low turbidity. Although light attenuation effects cannot be completely excluded, no direct measurements of medium absorbance or light penetration were performed. Therefore, the observed variations in pigment composition are primarily interpreted as metabolic responses to organic carbon supplementation rather than solely as optical shading effects.

The effect of FW supplementation was more pronounced in the production of phenolic compounds and antioxidant activity (Figure 3 and Figure 4). Total polyphenol content increased significantly at 1.5% FW, reaching approximately 8 µg mg^−1^ gallic acid equivalents (GAE) g^−1^ DW, which represents the highest value among all treatments (Figure 3). Statistical analysis confirmed significant differences between this condition and both the control and higher FW concentration treatments. Phenolic compounds are known to act as non-enzymatic antioxidants in microalgae, contributing to the neutralization of reactive oxygen species (ROS) generated during metabolic activity [34,35].

Consistent with the polyphenol results, DPPH radical scavenging activity also showed a marked enhancement in FW-supplemented cultures (Figure 4). The strongest antioxidant activity was again observed at 1.5% FW (~4.3 µmol Trolox equivalents g^−1^ DW), nearly doubling the activity measured in the control cultures. The increase in antioxidant activity observed at 1.5% FW should not be interpreted solely as an indicator of improved metabolic performance. Rather, it likely reflects an adaptive physiological response to moderate changes in cellular redox balance, where increased metabolic activity and organic carbon availability may enhance the formation of ROS, thereby stimulating the synthesis of antioxidant compounds. Conversely, the reduction in antioxidant activity observed at 3% FW suggests that higher organic loading does not further stimulate antioxidant defenses, but may instead lead to metabolic imbalance or reduced biosynthetic capacity. Under such conditions, excessive carbon availability may disrupt redox homeostasis beyond the adaptive capacity of the cells, resulting in a decline in measurable antioxidant compounds rather than their accumulation. These findings highlight that antioxidant responses in *L. platensis* are not linearly correlated with stress intensity, but instead reflect the balance between ROS generation and the cellular ability to activate protective mechanisms.

This limitation is also consistent with the broader challenges associated with maintaining stable microalgal cultures under high organic loads, where changes in medium properties and microbial dynamics can negatively affect biomass productivity [36].

Taken together, these responses suggest a biphasic effect of FW supplementation on *L. platensis*. At moderate concentration (particularly 1.5% FW), the FW appears to act predominantly as a beneficial mixotrophic substrate, supplying readily assimilable sugars that enhance metabolic activity and support the synthesis of antioxidant metabolites and proteins. In contrast, at higher supplementation (3% FW), the response is more consistent with indirect physiological stress than with demonstrated direct toxicity of a specific FW component. Under these conditions, excess organic load may alter redox homeostasis, increase respiratory pressure, and perturb carbon–nitrogen partitioning, thereby reducing the efficiency of pigment and PBPs biosynthesis. Since only the sugar fraction of FW was characterized in the present study, the inhibitory effects cannot be attributed to a single wastewater constituent, and are more cautiously interpreted as the result of overall compositional and metabolic stress.

It should be emphasized that the FW investigated in this study corresponds specifically to the washing and cleaning stages of dried fig processing. The composition of fig-processing effluents can vary substantially across the production chain, depending on factors such as thermal treatment, enzymatic processing, concentration steps, and the addition of formulation ingredients. As a result, the effects observed in this work cannot be directly extrapolated to all fig-derived WWs. In particular, downstream effluents may contain higher levels of degraded sugars, organic acids, or inhibitory compounds, which could lead to different microalgal responses. Therefore, the results presented here should be interpreted within the context of this specific wastewater stream, while highlighting its potential as a suitable substrate for mixotrophic cultivation.

The increase in antioxidant activity at intermediate FW concentrations may be associated with the activation of cellular defense mechanisms against oxidative stress generated during enhanced metabolic activity. Cyanobacteria respond to metabolic imbalance and ROS formation by producing antioxidant molecules such as phenolic compounds, carotenoids, and phycobiliproteins (PBPs) [35,37]. In mixotrophic systems, the simultaneous assimilation of organic and inorganic carbon sources can increase respiratory and photosynthetic fluxes, potentially elevating ROS formation and triggering protective biochemical responses.

A mechanistic explanation for these observations can be related to the sugar composition of dried figs, which is dominated by glucose and fructose, representing the primary soluble carbohydrates released during washing and processing operations (Table 2). These monosaccharides can be readily assimilated by cyanobacterial cells and metabolized through central carbon pathways, such as glycolysis and the oxidative pentose phosphate pathway. Under mixotrophic conditions, the simultaneous availability of organic carbon and photosynthetically derived energy enhances metabolic fluxes and biosynthetic activity. However, increased metabolic activity can also lead to elevated production of ROS, which in turn stimulates the synthesis of antioxidant molecules such as phenolics, carotenoids, and PBPs [38,39].

In addition to organic carbon, the potential contribution of mineral elements present in FW should also be considered when interpreting the observed responses. Although not quantified in this study, trace elements such as magnesium, iron, and zinc are known to play key roles in microalgal physiology, including chlorophyll biosynthesis, electron transport, and enzymatic activity. For instance, magnesium is a central component of chlorophyll molecules, while iron is essential for photosynthetic electron transport and PBP synthesis. Similarly, micronutrients such as zinc and manganese are involved in antioxidant enzyme systems [40]. Therefore, part of the observed variations in pigment content, antioxidant activity, and overall metabolic responses may not be solely attributable to organic carbon availability, but could also be influenced by the mineral composition of the FW. Future studies should include a detailed elemental characterization to better disentangle the relative contributions of organic and inorganic components.

The positive effect observed at 1.5% FW likely reflects an optimal balance between carbon availability and cellular metabolic capacity. At this concentration, organic substrates may enhance carbon assimilation and energy production without causing osmotic stress or metabolic inhibition. Conversely, higher concentrations of FW (3%) may introduce excessive organic compounds or inhibitory metabolites, leading to metabolic imbalance and reduced antioxidant activity. Similar patterns have been reported in microalgae cultivated with agro-industrial effluents, where moderate organic supplementation promotes metabolite synthesis while excessive loading negatively affects cellular physiology [26].

Overall, the results indicate that moderate supplementation of FW (1.5%) promotes the accumulation of antioxidant metabolites in *L. platensis* while maintaining stable levels of photosynthetic pigments. These findings highlight the potential of fig-processing effluents as sustainable substrates for microalgal cultivation, supporting the valorization of agro-industrial residues within a circular bioeconomy framework. The metabolic flexibility of *L. platensis* in utilizing organic substrates further confirms its suitability for integrated microalgal biorefineries aimed at producing biomass enriched in bioactive compounds. The comparison between mixotrophic and heterotrophic conditions further highlights that the positive effects of FW are strongly dependent on the presence of light, suggesting that FW acts primarily as a supplementary carbon source rather than a substitute for photosynthetic metabolism.

### 2.3. Proximate Biomass Composition of Limnospira platensis Under Salinity and Alkalinity

**Figure 5:**
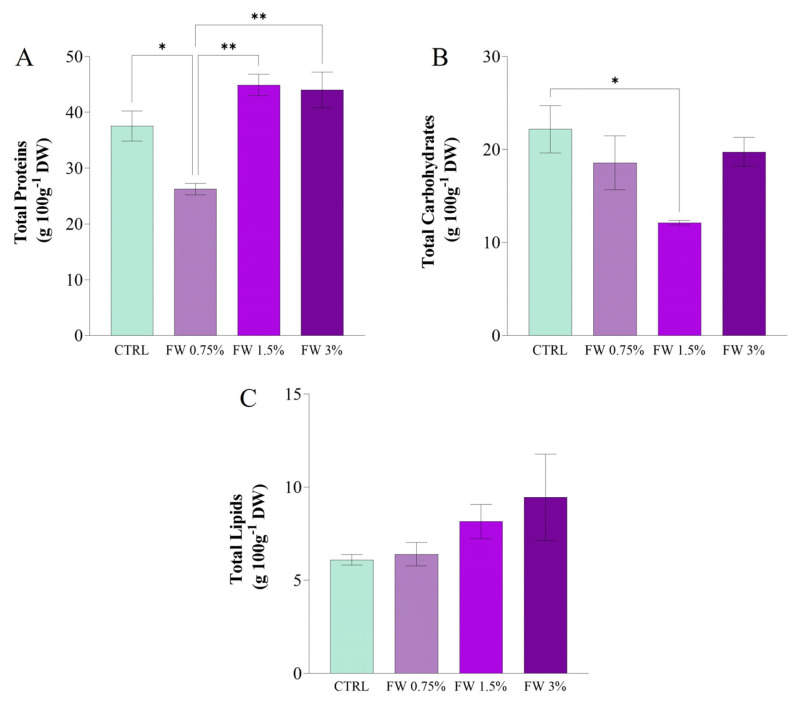
Biochemical composition of *L. platensis* biomass cultivated under mixotrophic conditions with different concentrations of fig wastewater (FW: 0.75%, 1.5%, and 3% *v v*^−1^). (**A**) Total proteins; (**B**) Total carbohydrates; (**C**) Total lipids. Values represent mean ± SD of three independent replicates. Mean differences were compared using Tukey’s test (*n* = 3, * *p* < 0.05, ** *p* < 0.01). In Figure 5C statistical analysis was performed using two-way ANOVA followed by Tukey’s post hoc test; no statistically significant differences were observed among treatments (*p* > 0.05).

The addition of FW as a supplement to the culture medium significantly affected the biochemical composition of *L. platensis*, specifically in terms of total proteins (TP), total carbohydrates (TC), and total lipids (TL) (Figure 5). These macronutrients are crucial for evaluating the metabolic state of the microalgae, as they reflect the synthesis of essential cellular components required for growth, stress responses, and storage.

TP content (Figure 5A) showed a significant increase in cultures supplemented with FW 1.5%, reaching approximately 48 g 100 g^−1^ DW, compared to the control (CTRL), which had a protein content of around 38 g 100 g^−1^ DW. Statistical analysis revealed significant differences between FW 1.5% and the other treatments, indicating that moderate organic carbon supplementation significantly enhanced protein synthesis. Proteins are critical for cellular metabolism and enzyme production, and the observed increase could be attributed to the enhanced nitrogen assimilation and metabolic activity triggered by the presence of readily available organic carbon [9,33]. This is consistent with findings that organic supplementation can stimulate nitrogen incorporation into proteins, as seen in other studies involving microalgae cultivated under mixotrophic conditions [26].

In contrast, TC content (Figure 5B) exhibited a decrease at 1.5% FW supplementation, with 19 g 100 g^−1^ DW, compared to the control and FW 3% conditions. Statistical analysis confirmed significant differences between FW 1.5% and the other FW treatments, suggesting that moderate organic carbon supplementation may redirect metabolic resources from carbohydrate storage to more immediate growth processes, such as protein synthesis. Similar observations have been made in microalgae, where mixotrophic conditions often reduce carbohydrate accumulation due to enhanced growth rates and the prioritization of nitrogen and protein metabolism [41]. At 3% FW, carbohydrate content recovered to a level comparable to the control, reflecting a possible shift in metabolic strategies as the organic carbon concentration increases.

TL content (Figure 5C) remained relatively stable across the experimental conditions, with no significant differences observed between the control and FW treatments. The lipid fraction ranged from 5 to 7 g 100 g^−1^ DW, indicating that while organic carbon availability (via FW supplementation) can influence the accumulation of proteins and carbohydrates, it does not significantly affect lipid synthesis under the conditions tested. This observation aligns with the idea that lipid metabolism in *L. platensis* is less responsive to moderate increases in organic carbon when compared to protein or carbohydrate metabolism [42].

Interestingly, the balance between protein and carbohydrate synthesis observed here differs from the typical chemical composition of *L. platensis*, which generally consists of 55–70% proteins, 15–25% carbohydrates, and 4–7% lipids [43]. This discrepancy may reflect the specific growth conditions and nutrient availability, which influence the partitioning of metabolic resources. The observed protein dominance under FW supplementation is consistent with the idea that microalgae, when supplied with organic carbon, often enhance protein biosynthesis due to increased metabolic activity [33].

The relative stability of lipid content in response to FW supplementation suggests that *L. platensis* may prioritize protein and carbohydrate synthesis when exposed to higher concentrations of organic carbon, as observed in other studies on microalgal responses to nutrient supplementation [9]. However, further studies are required to fully elucidate the intricate balance between these macronutrients under varying trophic conditions, especially in the presence of organic carbon sources such as FW.

The proximate composition data further support the interpretation of two concentration-dependent response regimes. The increase in protein content at intermediate FW suggests that moderate supplementation promoted biosynthetic activity under mixotrophic conditions. By contrast, the relative increase in lipid fraction at the highest FW concentration, together with the recovery of carbohydrate levels, is compatible with a shift toward stress-associated carbon reallocation rather than continued nutrient-driven enhancement. Thus, the compositional data are consistent with a transition from beneficial supplementation to metabolic imbalance as FW concentration increases.

In conclusion, FW supplementation, particularly at the 1.5% concentration, promotes protein biosynthesis and modifies the carbohydrate metabolism of *L. platensis*, highlighting the potential of fruit-processing residues as sustainable organic carbon sources for microalgal cultivation. These findings suggest that moderate organic carbon supplementation can enhance the growth and protein yield of *L. platensis*, which may have applications in biorefinery processes where high-value compounds such as proteins are of interest.

From a biotechnological perspective, the increase in protein content observed under FW supplementation is particularly relevant. *L. platensis* is widely recognized as a high-protein biomass suitable for applications in food, feed, and nutraceutical sectors, where protein-rich microalgal biomass represents a growing market segment. Compared to high-value pigments such as PC, bulk protein production is typically associated with larger-volume, lower-cost markets, but offers greater scalability and process robustness. Therefore, the enhancement of protein content under moderate FW supplementation suggests a potential shift toward protein-oriented biorefinery strategies, where biomass productivity and nutritional quality are prioritized over the extraction of single high-value compounds.

### 2.4. Phycocyanin Production by Limnospira platensis Cultivated in Fig Wastewater

**Figure 6:**
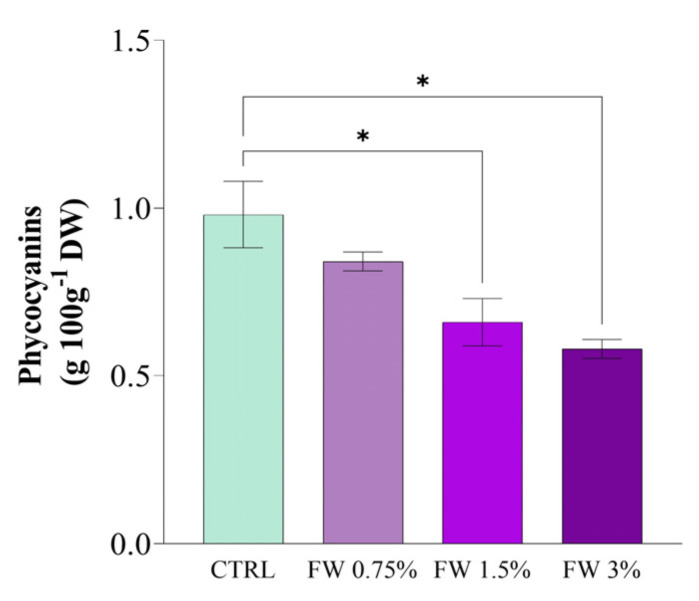
Phycocyanin content in *L. platensis* biomass cultivated under mixotrophic conditions with different concentrations of fig wastewater (FW: 0.75%, 1.5%, and 3% *v v*^−1^). Values represent mean ± SD (*n* = 3). Mean differences were compared using Tukey’s test (*n* = 3, * *p* < 0.05).

Phycocyanin (PC) production, a key pigment in *L. platensis* that holds potential applications as a natural colorant and antioxidant, was strongly affected by FW supplementation. As shown in Figure 6, the PC content of the biomass was strongly modulated by the concentration of FW added to the medium. However, the highest PC content and purity were consistently observed in the control condition (CTRL), with all FW treatments resulting in equal or lower performance. The highest PC content, 1.12 g 100 g^−1^ DW, was observed in cultures grown in CTRL. This was followed by cultures supplemented with 0.75% FW, where PC content decreased to 0.95 g 100 g^−1^ DW. The lowest PC levels were recorded at 1.5% and 3% FW, with values of 0.91 g 100 g^−1^ DW and 0.88 g 100 g^−1^ DW, respectively. Statistical analysis confirmed significant differences between the CTRL and FW 0.75%, suggesting that a low concentration of organic carbon promotes better pigment accumulation. Among the FW-supplemented cultures, 0.75% FW represented the most favorable condition; however, it did not surpass the CTRL, indicating that FW supplementation does not enhance PC production under the tested conditions. Interestingly, the addition of higher FW concentrations (1.5% and 3%) appeared to suppress PC synthesis, which contrasts with previous studies reporting enhanced PBP accumulation under mixotrophic conditions in *Arthrospira platensis*, depending on the cultivation regime and environmental parameters [44].

The progressive decline in PC content with increasing FW concentration indicates that PBP biosynthesis is more sensitive to FW supplementation than overall biomass production or total protein accumulation. This trend suggests that higher FW concentrations do not simply provide additional assimilable substrate, but rather shift the culture toward a less favorable physiological state for pigment synthesis [45].

At present, the available data do not allow the inhibitory effect to be attributed to a specific fig-derived compound. Instead, the observed response is more consistently explained by an indirect physiological stress associated with excessive organic loading. Under such conditions, increased carbon availability may alter intracellular redox balance and carbon flux distribution, affecting nitrogen allocation and ultimately limiting PBP biosynthesis. In this framework, moderate FW supplementation primarily acts as a nutrient source that supports metabolic activity, whereas higher concentrations appear to induce metabolic imbalance, potentially due to redox perturbation or the presence of uncharacterized soluble components in the effluent matrix.

These findings indicate that, although FW supplementation can sustain biomass growth and modulate other biochemical parameters, PC biosynthesis in *L. platensis* remains optimized under photoautotrophic conditions and is negatively affected by increasing organic carbon availability. This behavior reflects the intrinsic sensitivity of PBP synthesis to cellular metabolic balance, where excess organic carbon can disrupt redox homeostasis and reduce the allocation of resources toward light-harvesting pigments [46]. Therefore, FW supplementation cannot be considered advantageous for PC production per se, but rather as a factor that modulates the metabolic profile of the biomass depending on the target compound, highlighting the need to tailor cultivation strategies according to the desired biochemical output.

Additionally, variations in micronutrient availability, particularly iron, may also contribute to changes in PC biosynthesis, given its role in photosynthetic and pigment-related processes.

Previous studies indicate that PC production is driven by a complex interplay of factors, including the composition of the growth medium, the presence of organic carbon sources, and the physiological responses of microalgae to specific culture conditions, such as the appropriate addition of a nitrogen source. These conditions create a stress environment that can enhance PBPs synthesis [47]. However, in the present study, the mixotrophic cultures did not exhibit a notable rise in total PBP concentrations compared to the photoautotrophic control.

This outcome contrasts with earlier findings in *L. platensis* cultivated mixotrophically using cheese whey [48] and buttermilk waste [7] as an organic carbon source, where a positive correlation between organic load and PBP content was demonstrated. Similarly, higher PC levels compared to those in the conventional control Zarrouk medium were reported for *L. platensis* cultivated in tofu wastewater under mixotrophic conditions [49], and for *Galdieria sulphuraria* grown in media containing buttermilk [50]. These discrepancies between our findings and those in the literature might be attributed to the specific composition of the FW used in this study. The presence of certain organic compounds or inhibitory metabolites in FW may negatively impact PBPs synthesis, especially at higher concentrations.

**Figure 7:**
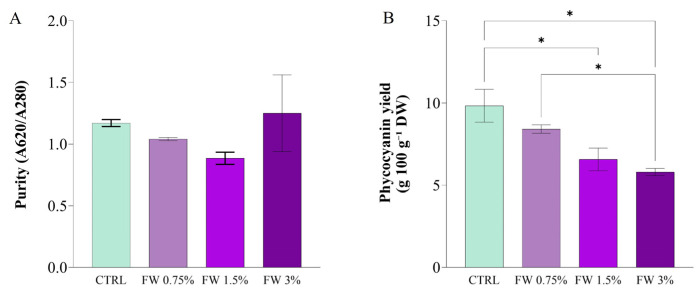
Phycocyanin extraction parameters from *L. platensis* cultivated under mixotrophic conditions with different concentrations of fig wastewater (FW: 0.75%, 1.5%, and 3% *v v*^−1^) (**A**) Phycocyanin purity (A620/A280); (**B**) Phycocyanin yield (g 100 g^−1^ DW). Values represent mean ± SD (*n* = 3). Mean differences were compared using Tukey’s test (*n* = 3, * *p* < 0.05).

PC purity (Figure 7A), assessed by the A620/A280 absorbance ratio, also showed significant variation across treatments. The highest purity value was observed in the CTRL cultures (A620/A280 = 1.5), followed by cultures supplemented with 0.75% FW (A620/A280 = 1.3). FW 1.5% and FW 3% treatments exhibited lower purity ratios, with values closer to 1.0, indicating the presence of more non-phycocyanin proteins and impurities in the extracts. These results suggest that moderate supplementation of organic carbon (0.75% FW) favors the accumulation of pure PC, while higher concentrations of FW may lead to the synthesis of other cellular components or the production of stress-related proteins that lower pigment purity. Similar observations have been made in studies where excessive organic carbon supplementation reduced pigment purity, likely due to the co-extraction of other cellular proteins and metabolites [7].

PC yield (Figure 7B), calculated as the product of concentration and biomass, followed a similar pattern to that observed for PC content. The highest PC yield was achieved in CTRL cultures (10.2 g 100 g^−1^ DW), while 0.75% FW supplementation increased the yield to 9.4 g 100 g^−1^ DW. Conversely, the 1.5% and 3% FW treatments resulted in significantly lower yields (7.2 g 100 g^−1^ DW and 6.5 g 100 g^−1^ DW, respectively). This trend is consistent with the hypothesis that moderate organic supplementation supports higher metabolic activity, which enhances PBP production, while excessive organic load reduces overall productivity due to metabolic inhibition or energy diversion to stress response pathways [33,51].

The purity of PC is heavily influenced by the extraction conditions used, including factors such as temperature, pH, solvent selection, biomass-to-solvent ratio, and the physical state of the biomass (fresh or dried) [52]. Efficient extraction techniques are essential for maximizing both yield and purity. Recent studies have shown that PC yield can be significantly enhanced by subjecting *Limnospira* biomass to specific abiotic stress conditions, such as freeze–thaw treatments combined with pulsed electric fields, which resulted in a remarkable increase in PC yield to 147.33 mg g^−1^ [53]. This suggests that optimizing environmental parameters, rather than exposing the biomass to extreme stress, can be an effective strategy for enhancing PC production without compromising the quality of the final product [54].

The market value of PC is closely tied to its purity level, with higher purity corresponding to significantly higher prices [55]. Analytical-grade PC, which typically has a purity level above 4, can command prices as high as 60 USD per gram, while premium-grade variants with even higher purity may reach extraordinary prices of up to 19.500 USD per gram. On the other hand, PC used in the cosmetics, food, and agrochemical industries is typically available at much lower prices, with biocolorants priced around 0.35 USD per gram and analytical-grade PC available at 4.500 USD per gram [56]. The disparity in prices is due to the complexities of PC extraction and purification, which remain the key factors driving the cost of this pigment. Projections indicate that the global PC market is expected to grow significantly, reaching 245.5 million USD by 2027, and further expanding to 279.6 million USD by 2030 [57]. This growth is fueled by the increasing demand for high-purity PC, particularly in niche markets such as natural colorants and functional food ingredients.

These findings demonstrate that FW supplementation modulates PC production in *L. platensis*; however, the highest PC content and purity were consistently observed in the control condition, with all FW treatments resulting in equal or lower performance. Among the supplemented cultures, 0.75% FW represented the most favorable condition, although it did not surpass the control. This indicates that, within the tested range, FW supplementation does not enhance PC production and that pigment biosynthesis remains optimized under photoautotrophic conditions.

The progressive decline in PC yield and purity observed at higher FW concentrations suggests that increasing organic carbon availability does not translate into improved pigment production, but rather imposes metabolic constraints. This behavior highlights the need to balance carbon availability with cellular metabolic capacity, as excessive organic supplementation may disrupt intracellular regulation and reduce the efficiency of phycobiliprotein biosynthesis.

From a process design perspective, these results have important implications. While photoautotrophic cultivation appears more suitable for maximizing PC output, FW-based mixotrophic systems may be better aligned with alternative valorization strategies. In particular, the reduced PC performance under FW supplementation should not be interpreted solely as a limitation, but rather as evidence of a shift in metabolic allocation toward other biomass components. Therefore, FW supplementation cannot be considered advantageous for PC production per se, but rather as a tool to modulate the biochemical profile of the biomass depending on the desired end-product, particularly in the context of protein-oriented or whole-biomass biorefinery applications.

## 3. Materials and Methods

### 3.1. Inoculums and Culture Media Preparation

The cyanobacterium *Limnospira platensis* SAG 21.99 used in this study was obtained from the Culture Collection of Algae at the University of Göttingen (SAG, Göttingen, Germany). The strain was maintained as a non-axenic culture under laboratory conditions. Cells were cultivated in a modified Jourdan medium (JM). The medium composition (g L^−1^) was as follows: NaHCO_3_ (5), KOH (1.6), NaNO_3_ (5), CaCl_2_·2H_2_O (0.027), K_2_SO_4_ (0.4), K_2_HPO_4_ (2), NaCl (1), MgSO_4_·7H_2_O (0.4), EDTA-Na_2_ (0.16), and FeSO_4_·7H_2_O (0.01). The medium was supplemented with 1 mL L^−1^ of a trace elements solution. The trace elements solution contained (mg L^−1^): EDTA-Na_2_ (250), H_3_BO_3_ (57), ZnSO_4_·7H_2_O (110), MnCl_2_·4H_2_O (25.3), CoCl_2_·6H_2_O (8.05), CuSO_4_·5H_2_O (7.85), and (NH_4_)_6_Mo_7_O_24_·4H_2_O (5.5). For inoculum preparation, 150 mL Erlenmeyer flasks containing 50 mL of JM were inoculated with approximately 10 mL of actively growing culture. The flasks were closed with sterile cotton plugs to allow gas exchange and maintained under continuous illumination provided by white fluorescent lamps (T8 36 W IP20, CMI, Wipperfürth, Germany). The incident light intensity was approximately 50 µmol photons m^−2^ s^−1^, measured using a luxmeter (HD 2302.0, Delta OHM, Padua, Italy). Cultures were maintained at room temperature. The inoculum cultures were grown for approximately one week, until reaching the late exponential growth phase, and were subsequently used to initiate the experimental cultivations.

### 3.2. Fig Processing Wastewater

Wastewater derived from the washing and cleaning stages of dried fig processing was collected from the agro-industrial facility “Mirò” located in San Mauro Cilento (SA), Italy. This stream represents an early-stage effluent, primarily composed of water-soluble sugars and low-molecular-weight organic compounds released from the fruit surface, and is characterized by minimal thermal or chemical processing compared to downstream effluents generated during concentration or formulation steps. An overview of the main sugars contained in the fig processing wastewater (FW) is presented in Table 2.

The chemical characterization of FW focused primarily on organic parameters (e.g., sugars, pH, total solids), while the concentration of mineral elements was not determined. It should be noted that trace elements such as Mg, Fe, and Zn may be present in fig-derived effluents and could potentially influence microalgal metabolism. This choice was motivated by the objective of the present study, which was to evaluate the effect of organic carbon supplementation on mixotrophic metabolism. Moreover, FW was used at relatively low concentrations (0.75–3% *v v*^−1^) in a nutritionally complete medium, which already provides the essential macro- and micronutrients required for the growth of *L. platensis*. Therefore, the contribution of mineral elements from FW is expected to be secondary under the tested conditions. Nevertheless, the potential influence of trace elements cannot be excluded and is acknowledged as a limitation of the present work. Future studies will incorporate a detailed elemental characterization of FW to better distinguish the respective contributions of organic and inorganic components.

After collection, the WW was stored at 4 °C until further use. It should be noted that storage at 4 °C and subsequent autoclaving may induce partial modification of the wastewater composition, including potential microbial degradation or thermal transformation of organic compounds. In this study, the wastewater was used under these practical conditions; therefore, the observed results reflect its effective composition during application rather than its original untreated state. Prior to cultivation experiments, the effluent was filtered through glass microfiber filters (GF/CTM, 47 mm diameter, Whatman, Incofar Srl, Modena, Italy) to remove suspended solids. The filtered WW was then sterilized by autoclaving at 121 °C and 0.1 MPa for 20 min before being used as an organic supplement for microalgal cultivation.

### 3.3. Cultivation Conditions and Experimental Setup

Cultivation experiments were conducted to evaluate the effect of FW as an organic carbon source on the growth and metabolic composition of *L. platensis* under mixotrophic and heterotrophic conditions. Heterotrophic experiments were performed to evaluate the ability of this cyanobacterium to utilize FW as a sole carbon and energy source in the absence of light, thereby providing a reference condition to interpret mixotrophic responses.

Experiments were performed in 500 mL Erlenmeyer flasks with a working volume of 300 mL. The flasks were closed with sterile cotton plugs to allow gas exchange and maintained at room temperature throughout the experiments. Mixing and gas exchange were ensured by gentle bubbling of sterilized air introduced through a sterile plastic cannula, connected to a compressed air source and filtered prior to entering the culture medium. This aeration system provided sufficient agitation to maintain biomass suspension and prevent sedimentation during cultivation. Magnetic stirring was not used, in order to maintain simple cultivation conditions and avoid potential mechanical stress on the filamentous biomass. In addition to ensuring mixing, aeration also facilitated gas exchange and CO_2_ availability, which is particularly important for cyanobacterial cultivation, as carbon supply contributes to maintaining the carbonate equilibrium of the culture medium and supports photosynthetic carbon fixation under illuminated conditions. Temperature was maintained at ambient laboratory conditions (approximately 25 °C) and remained stable throughout the experimental period. Dissolved oxygen was not directly monitored; however, continuous aeration with filtered air ensured efficient gas exchange and prevented oxygen limitation, maintaining conditions close to atmospheric equilibrium. The combination of aeration and passive temperature control provided stable cultivation conditions across all experimental setups.

To investigate the effect of organic carbon supplementation, sterilized FW was added to the culture medium at three different concentrations: 0.75% (*v v*^−1^) FW, 1.5% (*v v*^−1^) FW, and 3% (*v v*^−1^) FW. A control culture grown in standard medium without FW supplementation (CTRL) was included for comparison.

Two cultivation modes were investigated. In the mixotrophic experiments, cultures were exposed to continuous illumination (24 h light) provided by white fluorescent lamps delivering an incident light intensity of approximately 200 µmol photons m^−2^ s^−1^, allowing cells to simultaneously utilize inorganic carbon through photosynthesis and organic carbon supplied by the FW. In the heterotrophic experiments, the same FW concentrations were tested under dark conditions. To prevent light exposure and suppress photosynthetic activity, the cultivation flasks were completely wrapped with aluminum foil, forcing the cells to rely exclusively on the organic carbon present in the FW as the main carbon and energy source. The selected FW concentrations were chosen to represent low, intermediate, and relatively high levels of organic carbon supplementation, while avoiding excessive organic loading that could negatively affect culture stability.

All experimental conditions were performed in triplicate. Cultures were inoculated with actively growing *L. platensis* biomass to obtain an initial concentration of approximately 0.1 g L^−1^. The cultivation experiments lasted three weeks, and samples were collected daily to monitor microalgal growth through measurements of optical density and biomass concentration. At the end of the cultivation period, biomass was harvested and analyzed to determine final dry weight and biochemical composition, including pigments, antioxidant compounds, and macromolecular constituents.

### 3.4. Cell Growth and Dry Weight Determination

The growth of *L. platensis* cultures was monitored by measuring the optical density (OD) of the culture at 560 nm using a UV–Vis spectrophotometer (model ONDA V30 SCAN—UV VIS, ZetaLab, Padua, Italy). A calibration curve correlating OD values with dry biomass concentration was previously established and used to estimate biomass throughout the cultivation experiments. Dry biomass concentration was determined gravimetrically. Briefly, a known volume of culture (10 mL) was withdrawn from the cultivation flasks and filtered through pre-weighed glass microfiber filters (GF/CTM, 55 mm diameter, Whatman, Incofar Srl., Modena, Italy). Prior to filtration, the filters were dried in a forced-air oven (Memmert model 30, Memmert GmbH, Schwabach, Germany) at 105 °C for 2 h, cooled to room temperature in a desiccator, and weighed using an analytical balance (model M, Bel Engineering Srl, Monza, Italy) to obtain the initial filter weight (W_1_). After filtration, the retained biomass was dried overnight at 105 °C until constant weight and weighed again to obtain the final filter weight (W_2_). The dry biomass concentration was calculated based on the difference between the final and initial filter weights.

The final biomass concentration (dry weight), $X_{f}$ (g L^−1^), was calculated using the following equation:

(1) $$X_{f}=\frac{{\bar{W}}_{f}}{V_{s}}$$

where ${\bar{W}}_{f}$ represents the average dry biomass weight (g) obtained from triplicate sample at final cultivation time, and $V_{s}$ is the volume of culture sample (L) used for the analysis.

The biomass increase during cultivation (∆X) was calculated as:

(2) $$∆X=X_{f}-X_{0}$$

where $X_{f}$ is the final biomass concentration (g L^−1^) measured at time $t_{f}$ and $X_{0}$ is the initial biomass concentration (g L^−1^) measured at time $t_{0}$.

The average volumetric biomass productivity ($Q_{X}$) was determined as:

(3) $$Q_{X}=\frac{X_{f}-X_{0}}{t_{f}-t_{0}}=\frac{∆X}{∆t}$$

where ∆t represents the cultivation time (days).

The average specific growth rate ($\mu_{av}$) was calculated using the following equation:

(4) $$\mu_{av}=\frac{ln\left(\frac{X_{f}}{X_{0}}\right)}{t_{f}-t_{0}}=\frac{ln\left(\frac{X_{f}}{X_{0}}\right)}{∆t}$$

where $X_{f}$, $X_{0}$, $t_{f}$ and $t_{0}$ correspond to the final and initial biomass concentration and cultivation times, respectively.

The pH of culture suspensions was measured by a pH meter (model HI 2210, Hanna Instruments, Woonsocket, RI, USA).

### 3.5. Analysis of Proximate Composition

#### 3.5.1. Protein Analysis

Total protein content in *L. platensis* biomass was determined using a modified Lowry method [58]. Briefly, 2 mg of freeze-dried biomass were suspended in 5 mL of distilled water, and 0.5 mL of the resulting suspension was used for the assay. Prior to analysis, two reagents were prepared: Reagent A, consisting of 1% (*w v*^−1^) potassium sodium tartrate, and Reagent B, obtained by dissolving 2 g of sodium carbonate in 100 mL of 0.1 N sodium hydroxide. The working reagent was prepared by mixing 50 mL of Reagent B with 1 mL of Reagent A. For protein extraction and solubilization, the sample aliquot (0.5 mL) was mixed with 0.5 mL of 1 N NaOH and heated in a boiling water bath for 5 min. After cooling to room temperature, 2.5 mL of the working reagent were added and the mixture was allowed to react for 10 min. Subsequently, 0.5 mL of Folin–Ciocalteu reagent was added, and the reaction was allowed to proceed for 30 min in the dark at room temperature to ensure color development. Absorbance was measured at 750 nm using a UV–Vis spectrophotometer (Multiskan™ SkyHigh, Thermo Fisher Scientific Inc., Milan, Italy). Protein concentrations were calculated from a calibration curve prepared with bovine serum albumin (BSA) as the standard and expressed as BSA equivalents. All measurements were carried out in triplicate, and results are reported as mean ± standard deviation (SD).

#### 3.5.2. Lipid Analysis

Total lipid content was quantified following modified Bligh and Dyer [59] and Folch [60] extraction protocols. Approximately 2 mg of freeze-dried *L. platensis* biomass were treated with 1.5 mL of 1 N NaOH containing 25% methanol and incubated at 100 °C for 30 min to facilitate lipid release. After cooling, 3 mL of a methanol:chloroform mixture (1:2, *v v*^−1^) and 0.5 mL of 0.9% NaCl solution were added, and the suspension was vigorously vortexed. Phase separation was achieved by centrifugation at 4 °C for 10 min. The lower chloroform phase containing the extracted lipids was carefully collected. An aliquot (1 mL) of the chloroform fraction was evaporated under a nitrogen stream. The remaining residue was reacted with 100 µL of concentrated sulfuric acid, followed by the addition of 2.4 mL of 68% (*w v*^−1^) phosphovanillin reagent after thermal treatment. The mixture was incubated at room temperature for 10 min to allow color development. Absorbance was recorded at 530 nm using a spectrophotometer (Multiskan™ SkyHigh, Thermo Fisher Scientific Inc., Milan, Italy). Lipid concentration was calculated using an external calibration curve prepared with a 99% fat standard oil. All measurements were performed in triplicate, and results are expressed as mean ± standard deviation (SD).

#### 3.5.3. Carbohydrate Analysis

Total carbohydrate content was determined according to the phenol–sulfuric acid method described by Dubois et al. [61], with minor modifications. Briefly, 2 mg of freeze-dried biomass were suspended in 5 mL of distilled water. An aliquot of 0.2 mL of this suspension was transferred to a reaction tube and mixed with 0.2 mL of a 5% (*w v*^−1^) phenol solution. The reaction was initiated by the rapid addition of 1 mL of concentrated sulfuric acid, followed by gentle mixing. The tubes were subsequently cooled in an ice-water bath to stabilize the reaction and allow color development. Absorbance was measured at 488 nm using a spectrophotometer (Multiskan™ SkyHigh, Thermo Fisher Scientific Inc., Milan, Italy). A glucose standard solution was used to generate the calibration curve, and carbohydrate concentrations were expressed as glucose equivalents. All analyses were conducted in triplicate, and results are reported as mean ± standard deviation (SD).

### 3.6. Determination of Photosyntethic Pigment’s Content

Photosynthetic pigments were quantified following ethanol extraction of the biomass. Briefly, 2 mg of freeze-dried *L. platensis* biomass were suspended in 1.5 mL of 70% (*v v*^−1^) ethanol, vortexed thoroughly, and incubated in a boiling water bath for 15 min to promote pigment extraction. After incubation, the samples were centrifuged at 4000 rpm for 5 min, and the clear supernatant was collected for spectrophotometric analysis. The absorbance of the extract was measured at 665 nm, 649 nm, and 470 nm using a UV–Vis spectrophotometer in order to determine the concentrations of chlorophyll a, chlorophyll b, and total carotenoids, respectively. Pigment concentrations were calculated using the equations proposed by Arnon et al. [62]:

(5) $$Chl_{a}\left(\frac{mg}{L}\right)=\frac{\left(13.95A_{665}-6.88A_{649}\right)V_{2}}{V_{1}}$$

(6) $$Chl_{b}\left(\frac{mg}{L}\right)=\frac{\left(24.96A_{649}-7.32A_{665}\right)V_{2}}{V_{1}}$$

(7) $$C_{carotenoid}\left(\frac{mg}{L}\right)=\frac{\left(1000A_{470}-2.05C_{a}-114.8C_{b}\right)V_{2}}{245V_{1}}$$

where $V_{1}$ represents the volume of the sampled *L. platensis* suspension, $V_{2}$ the volume of the pigment extract supernatant, and $A_{665}$, $A_{649}$, and $A_{470}$ correspond to the absorbance values measured at 665, 649, and 470 nm, respectively. $C_{a}$ and $C_{b}$ indicate the calculated concentrations of chlorophyll a and chlorophyll b. It should be noted that *L. platensis*, being a cyanobacterium, naturally contains chlorophyll a as the primary photosynthetic pigment and does not synthesize chlorophyll b. Consequently, absorbance signals detected at 649 nm and mathematically attributed to chlorophyll b may arise from spectral overlap, pigment degradation products, or other accessory pigments. The values calculated using the standard equations were therefore retained only for comparative purposes with previously published spectrophotometric methods.

### 3.7. Determination of Phycocyanin Content

The extraction of phycobiliproteins (PBPs) was carried out using an aqueous saline solution as reported by Herrera et al. [63]. In details, a known amount (10 g) of frozen *Spirulina* biomass was put in 50 mL of an aqueous buffer solution containing 1% calcium dichloride (10 g L^−1^), frozen and thawed until complete cells break up, and stirred for 30 to 45 min. This extraction step was carried out twice and the obtained phycobilins solution was than separated by centrifugation at 8000 rpm for 10–15 min. The collected blue supernatant was used to perform optical readings on a spectrophotometer. The evaluation of different PBPs concentration, such as C-phycocyanin (PC), allophycocyanin (APC), and phycoerythrin (PE), was carried out by measuring the absorbance of each extract at three different wavelengths, 565 nm, 620 nm and 650 nm. The concentration of these PBPs, as mg mL^−1^ extract, was then determined from the equations established by Bryant et al. [64].

(8) $$\left[PC\right]=\frac{A_{620}-0.72A_{652}}{6.29}$$

(9) $$\left[APC\right]=\frac{A_{652}-0.191A_{620}}{5.79}$$

(10) $$\left[PE\right]=\frac{A_{565}-2.41\left[PC\right]-1.40[APC]}{13.02}$$

The concentration of total PBPs (mg mL^−1^) was determined as the sum of PE, PC, and APC in mg mL^−1^ of the extracted supernatant as follows:

(11) $$\left[PBPs\right]=\left[PC\right]+\left[APC\right]+\left[PE\right]$$

The PBP yield, estimated by relating the concentrations (expressed in terms of mg mL^−1^) to the biomass of *Spirulina* used (in terms of mg of dry weight), was obtained as follows:

(12) $$PBP\;\left(\frac{mg}{g}\right)=\frac{\left[PBPs\right]\;V_{ext}}{W_{b}.0.1}$$

where V_ext_ (mL) is the volume of the extract and W_b_ (g) is the weight of wet biomass undergone to the extraction procedure. In this equation, based on the experimental observations and literature information, the biomass pellet undergone to extraction is considered to have a solid content of 10%.

The phycocyanins (PC and APC) purity was calculated according to the following equations:

(13) $$PC\_purity\;(/)=\frac{\;A_{620}}{A_{280}}$$

(14) $$APC\_purity\;(/)=\frac{\;A_{650}}{A_{280}}$$

### 3.8. Determination of Antioxidant Activity (DPPH Assay)

The antioxidant activity of *L. platensis* biomass was evaluated using the DPPH radical scavenging assay, following a modified version of the method described by Brand-Williams et al. [65]. For extraction, approximately 2 mg of lyophilized algal biomass were suspended in 1 mL of ethanol and subjected to ultrasonic treatment for 1 min at 80% amplitude using a Bandelin Sonoplus HD 4100 ultrasonic probe (Bandelin Electronic GmbH & Co. KG, Berlin, Germany). The resulting suspension was centrifuged at 4000 rpm for 5 min at 20 °C, and the supernatant was collected for analysis. For the antioxidant assay, 50 μL of the extract or Trolox standard solution were mixed with 2 mL of a methanolic DPPH solution (40 μM). The reaction mixtures were incubated at room temperature for 60 min in the dark to allow radical scavenging. Following incubation, the absorbance was measured at 517 nm using 1 cm disposable cuvettes in a UV–Vis spectrophotometer (Multiskan™ SkyHigh, Thermo Fisher Scientific Inc., Milan, Italy). Antioxidant capacity was determined using an external calibration curve prepared with Trolox as the reference standard. The results were expressed as Trolox equivalent antioxidant capacity (TEAC), reported in mM g^−1^ of dry biomass. All measurements were performed in triplicate, and the results are presented as mean ± standard deviation (SD).

### 3.9. Determination of Total Polyphenol Content

The total polyphenol content of *L. platensis* biomass was determined using the Folin–Ciocalteu colorimetric assay, following a modified procedure based on Singleton et al. [66]. For extraction, 2 mg of freeze-dried biomass were suspended in 1 mL of ethanol–water solution (70:30, *v v*^−1^). From this extract, 100 μL were transferred into a reaction tube and mixed with 500 μL of Folin–Ciocalteu reagent. The mixture was allowed to react for 5 min at room temperature. Subsequently, 3 mL of sodium carbonate solution (10%, *w v*^−1^) were added, and the reaction volume was adjusted to 10 mL with ultrapure water. The mixture was incubated at room temperature for 90 min to allow complete color development. Absorbance was measured at 725 nm against a reagent blank using 1 cm disposable cuvettes in a Multiskan™ SkyHigh spectrophotometer (Thermo Fisher Scientific Inc., Milan, Italy). Quantification was performed using an external calibration curve prepared with gallic acid as the reference standard, and results were expressed as mg of gallic acid equivalents per gram of dry biomass (mg GAE g^−1^ DW). All measurements were carried out in triplicate, and the results are reported as mean ± standard deviation (SD).

### 3.10. Statistical Analysis

All experimental data related to *L. platensis* growth, pigment content, and biochemical composition were statistically analyzed using GraphPad Prism software (version 9.0, GraphPad Software, San Diego, CA, USA). A two-way analysis of variance (ANOVA) followed by Tukey’s multiple comparison test was applied to evaluate the influence of the experimental factors on the measured parameters. Differences between treatments were considered statistically significant at a 95% confidence level. Significance levels are indicated by asterisks according to the corresponding *p*-values, as follows: *p* > 0.05 (not significant), *p* < 0.05 (*)*, p <* 0.01 (**)*, p <* 0.001 (***), and *p* < 0.0001 (****). All experimental conditions were performed in triplicate, and the results are expressed as mean ± standard deviation (SD).

## 4. Conclusions

This study demonstrates that FW from early washing stages can be effectively used as an organic supplement for the mixotrophic cultivation of *L. platensis*, supporting a circular bioeconomy approach.

FW supplementation induced a concentration-dependent response. Moderate levels (≈1.5%) enhanced protein content and antioxidant activity, likely reflecting an adaptive metabolic response linked to redox balance. In contrast, higher concentrations (3%) led to metabolic imbalance, reducing pigment synthesis and overall biochemical efficiency.

PC production was not improved by FW, with highest yield and purity observed under control (photoautotrophic) conditions. This indicates a metabolic trade-off, where increased organic carbon shifts cellular resources away from pigment biosynthesis. Heterotrophic experiments confirmed that FW alone is insufficient to sustain efficient growth, reinforcing that photosynthesis remains essential, and that mixotrophy, not heterotrophy, is the viable strategy.

From an application perspective, FW supplementation is not suitable for PC-focused processes, but is promising for protein-rich biomass and whole-biomass valorization. Its integration should therefore be framed within a biorefinery approach, where multiple products contribute to process value.

From a process perspective, the use of FW as a supplementary substrate appears compatible with scale-up, particularly within integrated biorefinery frameworks. The availability of sugar-rich effluents from agro-industrial operations, combined with the ability of *L. platensis* to utilize organic carbon under mixotrophic conditions, supports the feasibility of implementing FW-based cultivation systems at larger scale. In this context, FW valorization may be particularly suited for protein-oriented production or for the generation of biomass enriched in antioxidant compounds and other bioactive molecules. However, further studies addressing process optimization, variability of wastewater composition, and large-scale operational stability are required to fully assess industrial applicability.

## Figures and Tables

**Table 1:** Effect of FW supplementation on *Limnospira platensis* growth kinetics parameters.

	*X_f_* (g L^−1^)	∆*X* (g L^−1^)	*Q_x_* (mg L^−1^ day^−1^)	*µ* (day^−1^)	*t_d_* (day)	pH
CTRL H	0.14 ± 0.04	0.09 ± 0.01	0.07 ± 0.01	0.62 ± 0.09	1.14 ± 0.16	9.65
FWH 0.75%	0.22 ± 0.00	0.14 ± 0.00	0.13 ± 0.01	0.86 ± 0.10	0.81 ± 0.09	9.70
FWH 1.5%	0.29 ± 0.01	0.21 ± 0.01	0.19 ± 0.01	1.09 ± 0.05	0.62 ± 0.04	9.71
FWH 3%	0.40 ± 0.02	0.32 ± 0.02	0.28 ± 0.03	1.25 ± 0.07	0.55 ± 0.02	9.64
CTRL M	1.99 ± 0.05	1.89 ± 0.05	0.29 ± 0.03	0.19 ± 0.02	3.68 ± 0.44	10.76
FWM 0.75%	1.97 ± 0.02	1.88 ± 0.01	0.30 ± 0.01	0.20 ± 0.01	3.41 ± 0.25	10.74
FWM 1.5%	2.10 ± 0.01	2.00 ± 0.00	0.31 ± 0.00	0.20 ± 0.00	3.50 ± 0.03	10.70
FWM 3%	2.02 ± 0.07	1.92 ± 0.06	0.31 ± 0.02	0.21 ± 0.01	3.33 ± 0.21	10.64

Note: *X_f_* = final biomass concentration, *Q_x_* = volumetric biomass productivity, Δ*X* = difference in average biomass concentration, *µ_av_* = average specific growth rate, *t_d_* = doubling time, CTRL H = control Jourdan medium under heterotrophy, FWH 0.75% = CTRL + 0.75% FW in heterotrophy, FWH 1.5% = CTRL + 1.5% FW in heterotrophy, FWH 3% = CTRL + 3% FW in heterotrophy, CTRL M = control Jourdan medium under mixotrophy, FWM 0.75% = CTRL + 0.75% FW in mixotrophy, FWM 1.5% = CTRL + 1.5% FW in mixotrophy, FWM 3% = CTRL + 3% FW in mixotrophy.

**Table 2:** Main sugars contained in the fig processing wastewater used in this study.

Total Sugars	Fructose	Glucose	Galactose	Lactose	Sucrose	pH
2.9	0.87	2.03	<LQ	<LQ	<LQ	5.3 ± 0.2

Note: the quantity of sugars is expressed in terms of g 100 g^−1^. <LQ = below the limit of quantification.

## Data Availability

Data will be available upon request.
